# Phytochemical basis and antitumor mechanisms of *Psoralea corylifolia* L.: implications for tumor immunomodulation and therapeutic development

**DOI:** 10.3389/fimmu.2026.1867059

**Published:** 2026-09-16

**Authors:** Yali Ren, Pengsheng Ye, Chen Cai, Li Zhu, Weishan Zhang, Zhixing Cao, Ting Sun

**Affiliations:** 1 Sichuan Academy of Agricultural Characteristic Plants, Chengdu, China; 2 State Key Laboratory of Southwestern Chinese Medicine Resources, School of Pharmacy, Chengdu University of Traditional Chinese Medicine, Chengdu, China

**Keywords:** anti-tumor mechanisms, hepatotoxicity, multidrug resistance, nano-delivery systems, phytochemistry, *Psoralea corylifolia* L.

## Abstract

**Background:**

*Psoralea corylifolia* L. (PCL), a medicinal plant used in Traditional Chinese Medicine for the treatment of “Kidney-Yang deficiency” has long been applied within a multi-component therapeutic framework. This integrative approach aligns with emerging oncological paradigms that emphasize network-based pharmacology; however, a comprehensive correlation between its phytochemical composition and anti-tumor activity remains insufficiently defined.

**Objective:**

This review aims to systematically evaluate the antineoplastic properties of PCL by integrating its phytochemical constituents with underlying pharmacological mechanisms and reported clinical relevance, thereby supporting its translational development.

**Methods:**

A comprehensive narrative review was performed using PubMed, Google Scholar, Web of Science, and CNKI to identify studies published from 2010 to August 2026. Evidence from *in vitro* systems, *in vivo* models, and clinical observations concerning the antitumor mechanisms, immunomodulatory effects, and safety profile of PCL was critically evaluated according to the experimental model and categorized by mechanistic domain.

**Results:**

The principal bioactive constituents, including coumarins, flavonoids, and monoterpenoids, demonstrated several anticancer effects. These compounds exerted synergistic effects through multiple mechanisms, such as promoting apoptosis and cell cycle arrest, inhibiting metastatic progression, attenuating multidrug resistance, modulating the tumor immune microenvironment, and altering cellular metabolism. Additionally, the balance between therapeutic efficacy and potential toxicity, particularly hepatotoxicity, is critically discussed, alongside current strategies, including nanotechnology-based delivery systems and combinatorial herbal approaches, designed to improve therapeutic performance.

**Conclusion:**

PCL represents a multi-target botanical candidate with potential utility in adjuvant cancer therapy. Future studies are required to clarify synergistic interactions, optimize delivery strategies, and support rigorous clinical translation.

## 1 Introduction

Cancer remains a major global public health burden, with persistently high morbidity and mortality rates (1). Recent estimates indicate that the number of new cancer cases worldwide will exceed 30 million by 2040, with a disproportionate increase in low- and middle-income countries (2). As the most populous country, China contributes approximately 24% of global incident cases and 30% of cancer-related deaths (3). Current cancer management relies primarily on surgery, radiotherapy, and chemotherapy (4), alongside rapidly evolving approaches such as targeted therapy and immunotherapy (4). However, tumor heterogeneity, the emergence of therapeutic resistance, and treatment-related toxicity severely limit therapeutic efficacy and negatively affect long-term patient outcomes (5, 6). Therefore, the identification of new anticancer agents derived from natural products, particularly those with multi-target activity and acceptable safety profiles, remains an important focus of drug discovery.

In this context, *Psoralea corylifolia* L. (PCL) has attracted increasing attention as a potential source of bioactive compounds. Phytochemical studies have systematically identified several major classes of constituents, including coumarins (e.g., psoralen and isopsoralen), flavonoids (e.g., corylin and bavachinin), and monoterpenophenols (e.g., bakuchiol) (7). Accumulating preclinical evidence indicates that these compounds exhibit anti-proliferative and pro-apoptotic effects in multiple cancer types, including breast, liver, and lung cancers.

Despite these advances, the available literature remains fragmented, and a comprehensive integration of the mechanistic evidence regarding PCL is still lacking. In particular, limited attention has been given to recently recognized pathways, such as immunogenic cell death, ferroptosis, and metabolic reprogramming. The present review therefore aims to provide an integrated overview of the phytochemical characteristics and anticancer mechanisms of PCL, linking traditional use with current molecular insights. By synthesizing evidence from intracellular signaling pathways to broader host-tumor interactions, this review aims to summarize the role of PCL-derived agents for oncology while also addressing relevant safety concerns, including hepatotoxicity. A graphical overview of the main topic covered in this review is provided in **Figure 1**.

**Figure 1:**
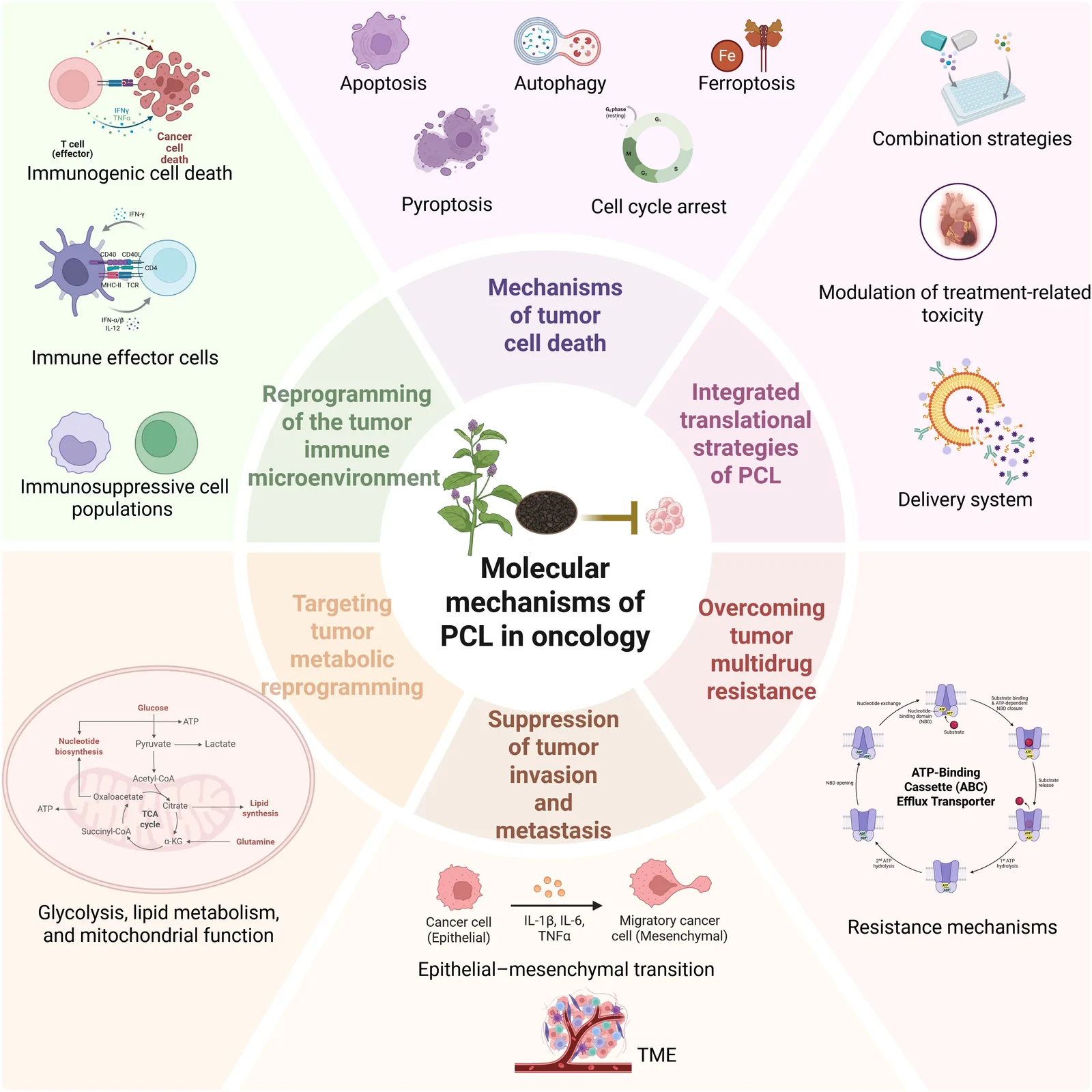
Graphical abstract. Created in BioRender. Leah, R. (2026) https://BioRender.com/4x9den8.

### 1.1 Literature search and selection criteria

This comprehensive narrative review was performed to synthesize current evidence on the phytochemical basis and antitumor mechanisms of PCL. No predefined review protocol or PRISMA-based approach was followed because this study was designed as a narrative review.

#### 1.1.1 Literature search strategy

A comprehensive literature search was performed using major scientific databases, including PubMed, Web of Science, Google Scholar, and CNKI. The search strategy combined controlled vocabulary (e.g., MeSH terms) and free-text keywords using the Boolean operators AND and OR. Additional sources included ClinicalTrials.gov, FDA approval information, major oncology guidelines, and reference lists of highly relevant reviews and primary studies. The primary search string covered the plant, its specific active compounds, the disease context, and broad pharmacological mechanisms: (“Psoralea corylifolia” OR “PCL” OR “psoralen” OR “bakuchiol” OR “isopsoralen” OR “corylin” OR “psoralidin” OR “psocorylins R” OR “astragalin” OR “angelicin”) AND (“tumor” OR “cancer” OR “carcinoma” OR “neoplasm”) AND (“mechanism” OR “immunomodulation” OR “tumor microenvironment” OR “metabolism”). The search primarily covered peer-reviewed articles published in English and Chinese from 2010 to August 2026, together with highly relevant seminal articles published before 2010.

#### 1.1.2 Study selection

All retrieved records were imported into a reference management system, and duplicate records were removed. Titles and abstracts were independently screened by two authors to identify relevant studies, followed by full-text assessment based on our developed eligibility criteria. Any disagreements arising during the screening process were resolved through discussion and consensus or, when necessary, consultation with a third author. Studies were selected based on their methodological rigor and relevance to the scope of this narrative review.

#### 1.1.3 Inclusion and exclusion criteria

Studies were included if they: (i) directly investigated the antitumor pharmacological mechanisms, immunomodulatory effects, or translational applications of PCL extracts or isolated PCL compounds; (ii) used *in vitro*, *in vivo*, or clinical models relevant to these mechanisms. Studies were excluded if they: (i) focused exclusively on agricultural, botanical, or analytical chemistry aspects; (ii) evaluated PCL exclusively as an inseparable component of complex traditional herbal formulas; (iii) were non-peer-reviewed sources, conference abstracts, or incomplete reports.

#### 1.1.4 Data synthesis and conceptual integration

Given the substantial heterogeneity in the experimental models, methods of active compound isolation, and mechanistic endpoints across the included studies, a quantitative meta-analysis was considered inappropriate. Therefore, a mechanistically oriented qualitative approach was used to synthesize the findings. The evidence was organized into key mechanistic domains, such as immunomodulation, tumor microenvironment regulation, and apoptosis, to illustrate how PCL exerts its systemic antitumor effects. A total of 151 articles were included in this review after the screening of the database results and the inclusion of essential background literature providing epidemiological and pharmacological context.

The included studies were categorized according to experimental model types to clarify the translational applicability of their findings, using the following classification criteria: (1) *in vitro*: studies performed using cultured cancer cell lines, which can elucidate basic molecular mechanisms at the cellular level but cannot recapitulate complex cell-cell interactions or an intact immune microenvironment; (2) *in vivo* syngeneic tumor models (conventional animal tumor models): tumor models established in immunocompetent animals. This platform retains an intact host immune system and is particularly suitable for investigating immunomodulatory effects, tumor microenvironment remodeling and immune-related antitumor mechanisms; (3) *in vivo* xenograft or patient-derived xenograft (PDX) models: models established using human-derived tumor tissues or cell lines. Such models preserve certain biological characteristics of human tumors but are generally established in immunodeficient mice, thereby limiting their applicability to the evaluation of immune-mediated antitumor effects; (4) in silico/computational analysis: network pharmacology, molecular docking, bioinformatic target prediction and other computational approaches that can identify potential targets and signaling pathways but require experimental validation and cannot alone provide definitive mechanistic evidence. Findings supported by two or more types of experimental models were classified as mixed multi-model evidence.

## 2 Ethnopharmacological heritage and its modern oncological interpretation

PCL has been extensively used in both Traditional Chinese Medicine (TCM) and Ayurveda (8, 9), with its applications documented in classical sources such as the *Compendium of Materia Medica (Ben Cao Gang Mu)* (10). Traditionally, its functions are summarized as “warming the kidney to reinforce Yang, directing qi downward to relieve wheezing, and warming the spleen to arrest diarrhea” (7). These concepts are based on the holistic framework of TCM, in which therapeutic interventions are intended to restore functional balance by supporting spleen and kidney Yang activity.

In contemporary biomedical terms, these traditional indications have been tentatively associated with systemic regulatory effects on the host. Advanced cancer is frequently accompanied by complex symptom clusters, including cancer-related fatigue, gastrointestinal dysfunction, and skeletal complications, which have been qualitatively compared to features described as “Yang deficiency” in TCM. In this context, the traditional use of PCL in strengthening bone and supporting systemic function has been explored as a potential rationale for its use in managing cancer-related complications, such as skeletal-related events (SREs) in metastatic disease. These observations suggest that the potential therapeutic role of PCL may extend beyond direct cytotoxic effects. Instead, its activity may involve modulation of the host physiological environment, including immune and metabolic processes, which could indirectly influence tumor progression and response to therapy. However, these associations remain largely conceptual and require further experimental and clinical validation.

## 3 Phytochemistry and pharmacodynamic basis of antitumor activity

The therapeutic effects of PCL are attributed to its chemically diverse composition, characterized by multiple bioactive constituents with potential complementary activities. To date, more than 40 bioactive compounds have been identified, mainly belonging to furanocoumarins, flavonoids, and monoterpenophenols. Although PCL exhibits several pharmacological properties, including anti-inflammatory and osteogenic effects, its antitumor activity has become a major focus of recent research (9). This section summarizes the principal chemical classes and their reported contributions to antitumor effects.

### 3.1 Furanocoumarins: major bioactive constituents

Furanocoumarins are considered key constituents of PCL and are commonly used as markers for quality control. Among them, psoralen and isopsoralen are the most extensively studied due to their relatively high abundance and interference with tumor cell homeostasis (11). These compounds have been shown to influence tumor cell proliferation by affecting cell cycle progression and apoptosis. In addition, derivatives such as psoralidin and 8-methoxypsoralen have demonstrated the ability to induce cell cycle arrest in various cancer models. Beyond direct cytotoxic effects, some furanocoumarins are increasingly investigated for their potential role in modulating multidrug resistance, although the underlying mechanisms remain to be fully clarified.

### 3.2 Flavonoids: structurally diverse bioactive compounds

Flavonoids represent the most structurally diverse group in PCL, including dihydroflavones (e.g., bavachin), isoflavones (e.g., corylin), and chalcones (e.g., isobavachalcone). In addition to their antioxidant and estrogen-like activities, these compounds are potential anticancer agents. Several studies suggest that PCL-derived flavonoids inhibit epithelial-mesenchymal transition, thereby reducing tumor cell migration, invasion, and metastatic potential (12). Moreover, the presence of prenyl groups in many of these molecules is associated with increased lipophilicity and improved cellular uptake, which may contribute to enhanced biological activity.

### 3.3 Monoterpenophenols: characteristic meroterpenoid compounds

Bakuchiol, a representative monoterpenophenol of PCL, is characterized by a meroterpenoid structure and has been widely studied for its biological activity (13). In addition to its known antioxidant properties, recent studies have explored its potential role in oncology. In particular, bakuchiol has been reported to enhance the sensitivity of cancer cells to certain chemotherapeutic agents. Furthermore, the identification of dimeric derivatives (e.g., bakuchiol A-U) expanded the chemical diversity of this class and may provide additional candidates for further investigation (14).

### 3.4 Auxiliary constituents and potential combinatorial effects

PCL also contains other components, including benzofurans (e.g., corylifonol), polysaccharides, and volatile oils (15). Although their independent antitumor effect is less well established compared to the major classes, these components may potentially contribute indirectly by influencing the tumor immune microenvironment or by affecting the bioavailability and intestinal absorption of other active compounds. Overall, the combined presence of multiple constituents may underlie the integrated pharmacological effects of PCL, although the extent and mechanisms of such interactions remain to be systematically investigated.

## 4 Molecular mechanisms of PCL in oncology

### 4.1 Mechanisms of tumor cell death

#### 4.1.1 Induction of apoptosis

Apoptosis represents the primary mechanism underlying the cytotoxic effects of PCL, involving mitochondrial death receptor and endoplasmic reticulum (ER) stress-related pathways, together with the modulation of survival signaling cascades (**Figure 2**).

**Figure 2:**
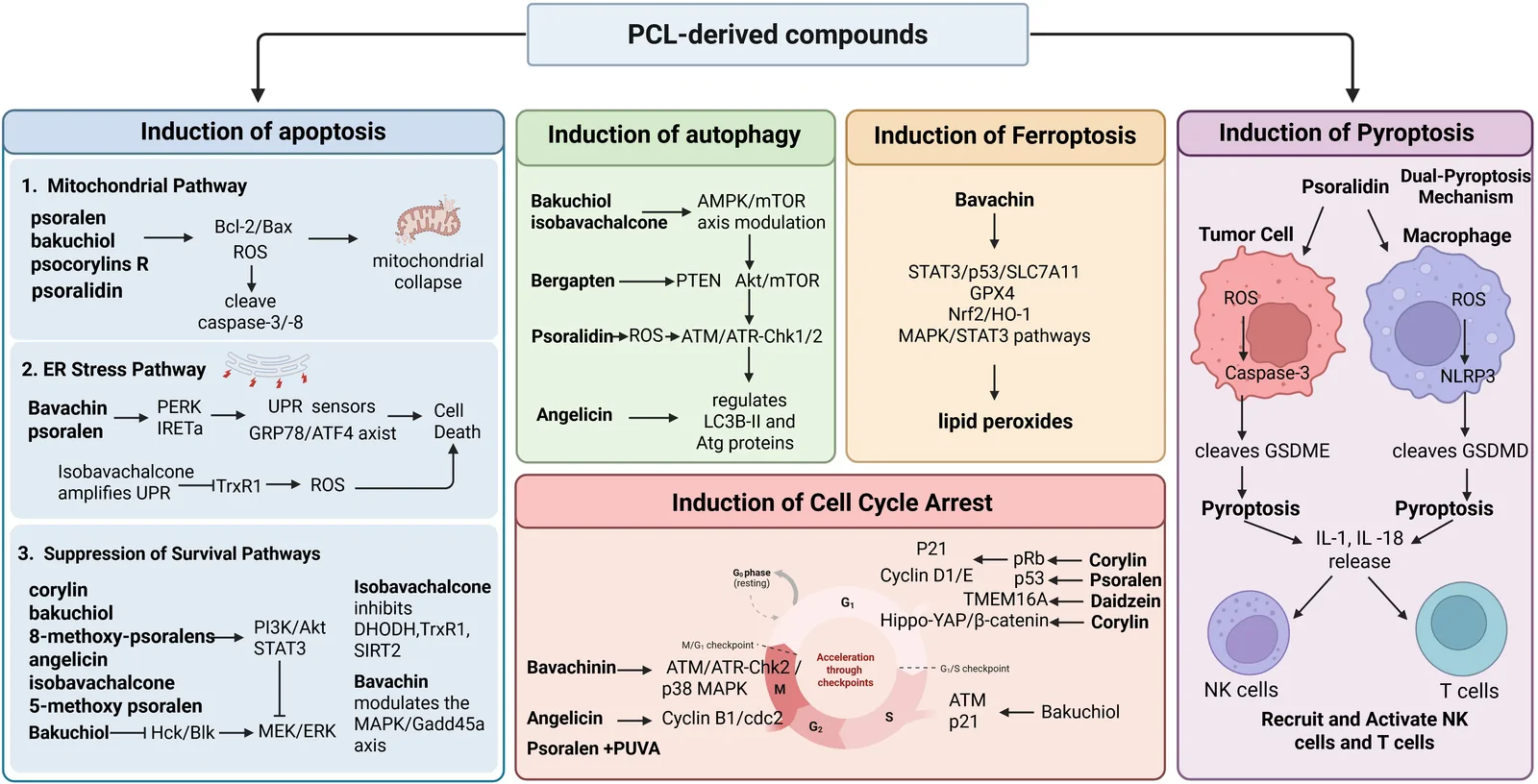
Mechanisms of tumor cell death. Created in BioRender. Leah, R. (2026) https://BioRender.com/66dd0lo.

PCL-derived compounds promote apoptosis in cultured cancer cell lines by regulating members of the Bcl-2 family and activating caspase-dependent pathways. For example, psoralen, bakuchiol, and psocorylin R alter the Bcl-2/Bax balance and activate caspase-3 and 8 in human hepatoma and breast cancer cells (12, 16–18). Oxidative and ER stress are also prominent triggers of apoptosis in these cell models. Psoralidin promotes mitochondrial dysfunction by increasing ROS levels (19–23). Similarly, astragalin possesses pro-apoptotic effects in melanoma (24) and colon cancer models (25). Angelicin induces apoptosis in squamous cell carcinoma through the modulation of the DUSP6-mediated c-MYC signaling pathway (26), while psoralen triggers p53-independent apoptosis in cutaneous T-cell lymphoma (27). Bavachin and psoralen activate unfolded protein response-related signaling, including IRE1α, PERK, and GRP78/ATF4 pathways, leading to CHOP-mediated cell death (17, 28–30). Isobavachalcone further enhances this stress response (31) while also interacting with ERK1/2 and RSK2 kinases, thereby blocking downstream survival signals and inducing apoptosis (32). Notably, apoptosis is the most extensively investigated mechanism across various cancer cell lines.

Multiple PCL-derived compounds modulate key pro-survival pathways, including PI3K/AKT and STAT3 signaling. Compounds such as corylin (33), bakuchiol (34), 8-methoxypsoralen (35, 36), angelicin (37, 38), isobavachalcone (39, 40), and 5-methoxypsoralen (bergapten) (41, 42) inhibit these pathways. Beyond direct induction of apoptosis, PCL components also suppress tumors by dysregulating pro-survival signaling networks. For example, bakuchiol is an ATP-competitive inhibitor of Hck and Blk, leading to the suppression of MEK/ERK and AKT/p70S6K signaling cascades (43). Isobavachalcone targets enzymes such as DHODH (44), TrxR1 (31), and SIRT2 (45), while bavachin regulates the MAPK/Gadd45a axis (46), highlighting the multi-target pharmacology of PCL. However, most of these signaling network interactions have been validated only *in vitro*, with limited systematic *in vivo* validation or pharmacokinetic characterization.

#### 4.1.2 Induction of autophagy

Autophagy is an additional mechanism contributing to the anticancer activity of PCL components. Current reports on this mechanism are exclusively based on *in vitro* models. For example, bakuchiol and isobavachalcone induce autophagy by modulating the AMPK/mTOR axis (47, 48), while bergapten, psoralidin, and angelicin promote autophagy through PTEN/Akt/mTOR signaling (49), ROS-dependent stimulation of the ATM/ATR-Chk1/2 pathway (50, 51), and the regulation of LC3B-II and Atg proteins (52), respectively.

#### 4.1.3 Induction of ferroptosis

Ferroptosis is an iron-dependent form of cell death characterized by lipid peroxidation. Certain PCL-derived flavonoids induce ferroptosis (53, 54). For instance, bavachin promotes ferroptosis in osteosarcoma cells by modulating the STAT3/p53/SLC7A11 axis, resulting in the downregulation of GPX4 (55). This compound is also associated with the disruption of antioxidant defense systems in liver and laryngopharyngeal cancers through the Nrf2/HO-1 and MAPK/STAT3 pathways (56, 57). In addition to direct cytotoxic effects, ferroptosis-associated lipid peroxidation products and damage-associated molecular patterns (DAMPs) influence immune cell responses, including macrophage activity; however, these effects require further investigation.

#### 4.1.4 Induction of pyroptosis

The pyroptotic effects of PCL are supported by *in vitro* and *in vivo* evidence. Psoralidin simultaneously activates Gasdermin E cleavage in tumor cells through the ROS/caspase-3 signaling and Gasdermin D cleavage in macrophages through the activation of the ROS/NLRP3 inflammasome pathway. These processes are associated with the release of proinflammatory cytokines, including IL-1β and IL-18, contributing to immune activation. However, the broader immunological implications of these effects remain to be fully elucidated (58).

#### 4.1.5 Induction of cell cycle arrest

PCL-derived compounds inhibit tumor cell proliferation by arresting the cell cycle at the G1/S or G2/M phases, with several key findings corroborated by murine models.

G1 phase arrest: This process is often associated with the upregulation of p21 and the downregulation of cyclin D1/E. Reported mechanisms include the modulation of pRb phosphorylation by corylin (33), p53 activation by psoralen (29), and inhibition of the TMEM16A channel by daidzein (59, 60). Angelicin influences the Hippo-YAP/β-catenin pathway, contributing to G1 arrest (61).

G2/M phase arrest: This phase is frequently associated with DNA damage responses. Bavachinin activates the ATM/ATR-Chk2 and p38 MAPK signaling pathways (62, 63), while angelicin reduces the expression of the cyclin B1/cdc2 complex (64). Furthermore, psoralen in combination with PUVA therapy inhibits G2/M transition (27).

S phase arrest and multi-target regulation: Bakuchiol induces S-phase arrest through the activation of ATM and upregulation of p21 (18, 65). Additionally, crude PCL extracts promote degradation of cyclin D1 and CDK4 through the ERK1/2/GSK3β pathway, suggesting multi-level regulatory effects (66).

### 4.2 Reprogramming of the tumor immune microenvironment

#### 4.2.1 Immunogenic cell death and antigen presentation

Immunogenic cell death (ICD) represents one of the mechanisms contributing to the activation of adaptive antitumor immunity primarily through the release of DAMPs from dying tumor cells, which promote dendritic cell (DC) maturation and antigen presentation. Astragalin induces ICD in hepatocellular carcinoma cells potentially through targeting NQO2 and promoting ROS-mediated ER stress, as demonstrated by *in vitro* and murine hepatoma models (67). This process is associated with the exposure of calreticulin on the cell-surface, as well as the extracellular release of ATP and high-mobility group box 1 (HMGB1), which collectively facilitate DC activation and subsequent T-cell priming. In addition to flavonoid-mediated ER stress, recent studies suggest that mitochondrial damage-related pathways also contribute to the activation of antitumor immune responses by PCL-derived compounds. For instance, isobavachalcone has been reported to directly target dihydroorotate dehydrogenase (DHODH), resulting in ROS accumulation, mitochondrial membrane remodeling, and subsequent activation of the STING pathway in preclinical models of gastric cancer (68).

The PD-1/PD-L1 axis is a major immune checkpoint pathway exploited by tumors to evade immune clearance. The interaction of PD-1 with the tumor-expressed PD-L1 attenuates TCR signaling and contributes to T cell exhaustion (69). Although PD-1/PD-L1 blockade has demonstrated clinical efficacy, response rates are limited in many cancer types because of primary and acquired resistance (70). Consequently, the identification of tumor microenvironment (TME)-modulating agents to overcome this resistance and sensitize tumors to immunotherapy is of considerable clinical interest (71). PCL-derived compounds may have immunomodulatory activity in this combination setting. For example, astragalin has been reported to enhance the antitumor efficacy of PD-L1 blockade in preclinical models (67).

#### 4.2.2 Modulation of immune effector cells

PCL-derived compounds influence both adaptive and innate immune responses, potentially contributing to antitumor activity through multiple mechanisms. CD8^+^ cytotoxic T lymphocytes are core mediators of adaptive antitumor immunity, whose tumor infiltration and functional activation directly determine the efficacy of antitumor immune response. However, chronic antigen stimulation and exposure to immunosuppressive factors, including IL-10 and TGF-β, secreted by M2-type TAMs, can cause CD8^+^ T cells to progressively lose their effector functions and enter a dysfunctional state known as T cell exhaustion (72). Exhausted T cells (Tex) exhibit a hierarchical loss of effector functions compared to effector or memory T cells, including a reduced capacity to produce IL-2 (a proliferative signal), TNF-α, and IFN-γ, the critical mediator for antitumor efficacy (73, 74).

PCL-derived compounds effectively counteract this exhaustion cascade. Bakuchiol and isobavachalcone significantly enhance CD8^+^ T-cell infiltration and the production of effector cytokines, including interferon-γ (IFN-γ) and tumor necrosis factor-α (TNF-α) in lung and pancreatic cancer models (75, 76). Psoralidin also modulates the TME by promoting IFN-γ production, which further reinforces the activation of adaptive antitumor immunity in mouse models of breast cancer (77).

In addition to adaptive immunity, innate immune cells are also affected. They represent the first line of antitumor defense, and their functional state shapes both the local TME and systemic antitumor immunity. Traditionally, activated macrophages have been conceptually categorized into classical M1 and alternative M2 phenotypes (78). M1 macrophages within the TME are classically viewed as exerting pro-inflammatory and tumor-suppressive functions by producing cytokines that activate cytotoxic immune cells. Conversely, macrophages in the TME often adopt an M2-like phenotype, associated with tumor-promoting functions, and these tumor-associated macrophages (TAMs) actively support tumor growth by secreting immunosuppressive factors, promoting angiogenesis, and remodeling the extracellular matrix (79, 80). Within this classical framework, bakuchiol promotes a shift in macrophage polarization from the protumor M2 phenotype toward the antitumor M1 phenotype, thereby enhancing the antitumor immune response (75). However, the binary M1/M2 classification oversimplifies the substantial heterogeneity and plasticity of TAMs (81). Since diverse macrophage activation states frequently coexist within the TME, how bakuchiol modulates this complex TAM population warrants further investigation. Furthermore, the combination of daidzein and exercise enhances the mobilization of natural killer (NK) cells from tissue reservoirs (e.g., the spleen) into the systemic circulation and tumor sites through an epinephrine/IL-6-dependent mechanism (82), although the specific contribution of PCL-derived compounds in this context requires further clarification.

#### 4.2.3 Regulation of immunosuppressive cell populations

Adaptive immunity, characterized by antigen-specific T- and B-cell responses and immunological memory, is a major component of the antitumor immune response (83). Within the cancer-immunity cycle, following the cross-presentation of tumor antigens by dendritic cells, naive CD8^+^ T cells are activated and differentiate into cytotoxic T lymphocytes (CTLs) (84). These CTLs subsequently recognize antigenic peptides presented by MHC class I molecules on tumor cells (85) and exert targeted cytotoxicity through the release of perforin and granzymes (86). However, this adaptive immune response is frequently constrained by diverse immunosuppressive cell populations within the TME (87).

Immunosuppressive cell populations, including regulatory T cells (Tregs) and myeloid-derived suppressor cells (MDSCs), are involved in TME immunosuppression and T cell dysfunction. Bakuchiol and isobavachalcone simultaneously reduce the presence of Tregs and MDSCs in the TME of lung and pancreatic tumor-bearing mice, thereby alleviating their inhibitory effects on effector immune cells (75, 76). Similarly, psoralidin influences Treg-associated immunosuppressive activity in breast cancer models, shifting the local immune balance from a protumor immunosuppressive state toward an antitumor phenotype (77).

Overall, these findings suggest that PCL-derived compounds may contribute to the modulation of the tumor immune microenvironment through multiple mechanisms, including the enhancement of tumor immunogenicity (through ICD and pyroptosis), regulation of immune effector cells (T cells and NK cells), and attenuation of immunosuppressive pathways (M2 repolarization), as shown in **Figure 3**. Notably, these immunomodulatory effects are supported by evidence from *in vivo* murine tumor models compared with many classical pathways that have been investigated primarily *in vitro*, highlighting their translational relevance. Nevertheless, most of these findings remain preclinical, and their clinical relevance requires validation in human studies.

**Figure 3:**
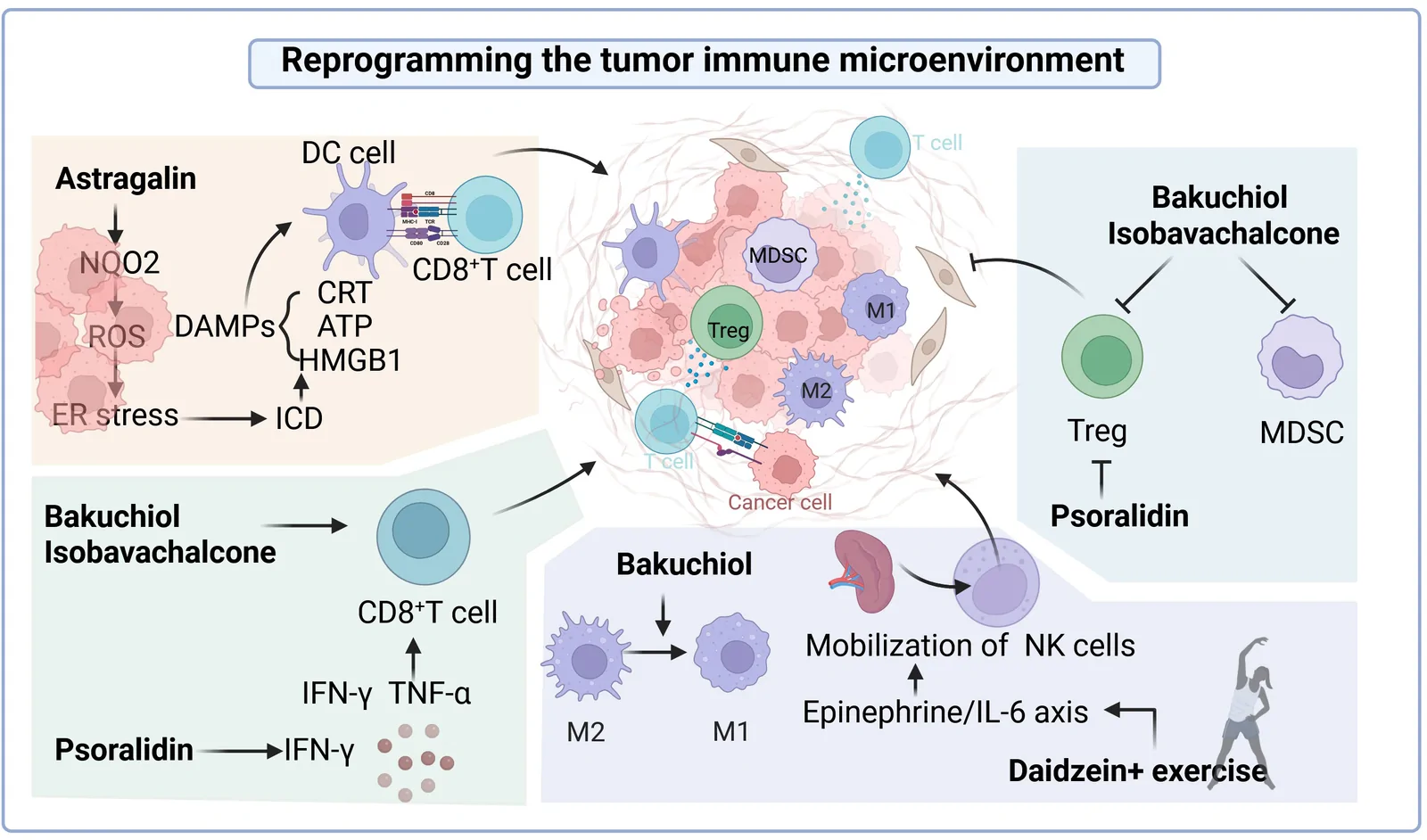
Reprogramming of the tumor immune microenvironment. Created in BioRender. Leah, R. (2026) https://BioRender.com/2370gp3.

### 4.3 Targeting tumor metabolic reprogramming

Tumor cells undergo metabolic reprogramming to sustain proliferation and meet increased demands for biosynthetic precursors and energy. In addition to enhanced glycolysis (Warburg effect), this process involves coordinated changes in glucose, lipid, amino acid, and nucleotide metabolism. Metabolic plasticity represents a key feature of tumor cells, as inhibition of one pathway (e.g., glycolysis) may lead to compensatory activation of oxidative phosphorylation (OXPHOS). In this context, certain PCL-derived compounds affect multiple metabolic pathways. For example, bergapten inhibits glycolytic activity, reduces lactate production, and interferes with mitochondrial respiratory function, potentially leading to decreased ATP generation (88). This suggests that bergapten interferes with central hubs governing global metabolic flux. In addition, lipid metabolism is an important target in tumor biology. Bergapten modulates the liver X receptor (LXR) pathway. In liver cancer models, it activates LXRα, leading to the upregulation of the cholesterol transporter ABCA1 and downregulation of SREBP1-mediated lipogenesis (89). These findings suggest a potential role for bergapten in regulating lipid homeostasis, although further validation is required.

Some PCL-derived compounds appear to preferentially affect aerobic glycolytic pathways. Astragalin reduces glucose uptake and utilization through the activation of the cellular energy sensor AMPK and inhibition of downstream mTORC1 signaling, leading to downregulation of glycolysis-related genes, including the glucose transporter GLUT1 and lactate dehydrogenase A (LDHA) (90). In hepatocellular carcinoma models, astragalin upregulates miR-125b, resulting in the inhibition of hexokinase 2 (HK2). This suppression promotes a metabolic shift toward mitochondrial OXPHOS, resulting in excessive ROS production and reduced tumor cell proliferation (91). However, the context-dependent nature of these effects should be considered.

Mitochondrial dysfunction represents another potential mechanism of action. The flavonoid bavachin induces mitochondrial membrane depolarization and alters calcium homeostasis, both impairing cellular bioenergetics and simultaneously inhibiting OXPHOS and glycolysis (28). The dependence of tumor cells on mitochondrial function for energy production and biosynthesis contributes to reduced cell viability. However, the relative contribution of these mechanisms compared to other pathways remains to be clarified. Overall, PCL-derived compounds influence tumor metabolism through multiple mechanisms, including modulation of glycolysis, lipid metabolism, and mitochondrial function, as shown in **Figure 4**. These findings suggest that metabolic regulation may contribute to the overall pharmacological effects of PCL. However, most of the available evidence is derived from preclinical studies, and further investigation is required to determine the clinical relevance of these observations.

**Figure 4:**
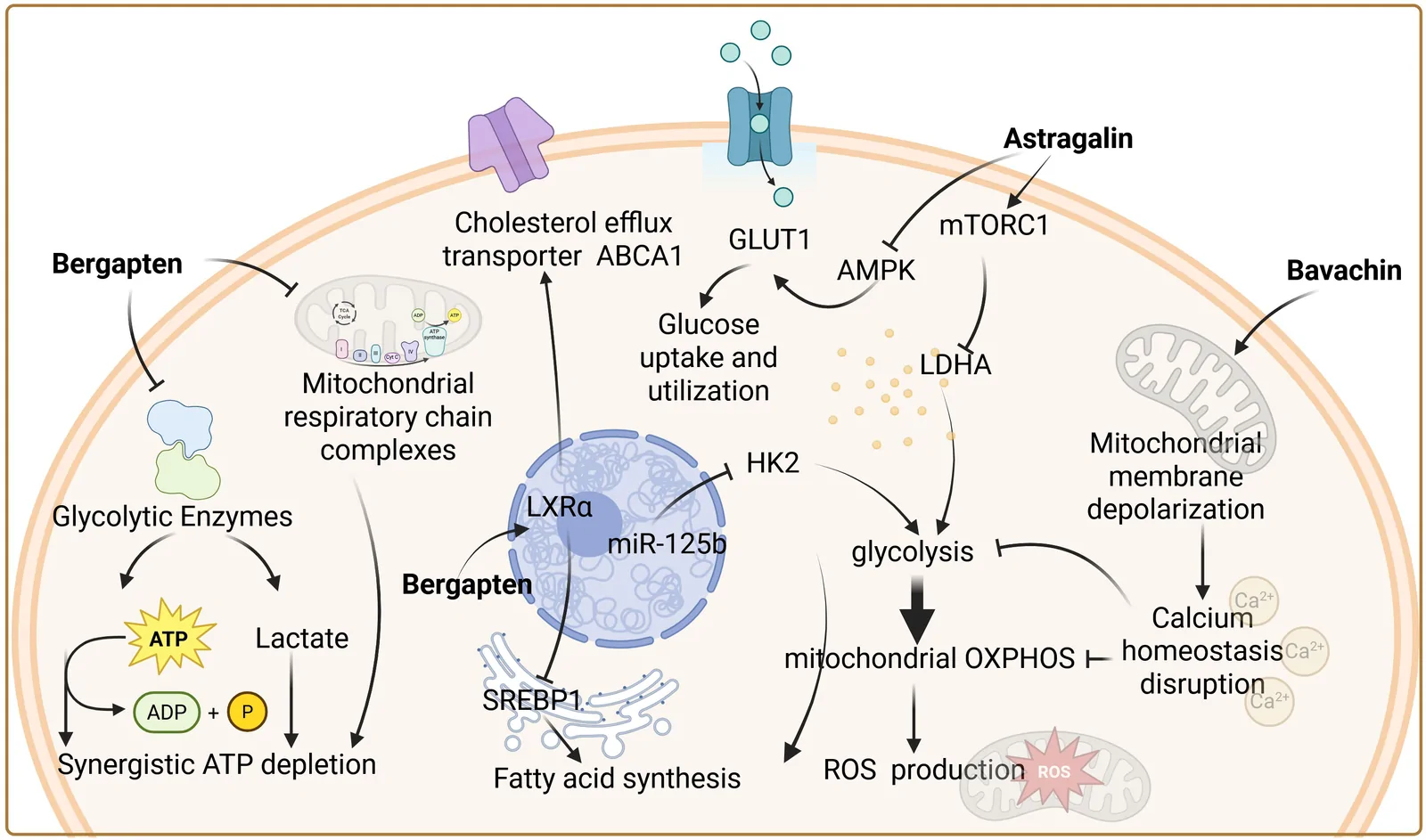
Targeting tumor metabolic reprogramming. Created in BioRender. Leah, R. (2026) https://BioRender.com/6l123xt.

### 4.4 Suppression of tumor invasion and metastasis

#### 4.4.1 Modulation of the epithelial–mesenchymal transition

Metastasis remains the main cause of cancer treatment failure and cancer-related mortality worldwide, involving the acquisition of invasive properties, immune evasion, and colonization of distant organs. EMT is a key biological process associated with enhanced migratory and invasive capacity of tumor cells, and is the initial step of the metastatic cascade. PCL-derived compounds influence EMT-related pathways, effectively reversing the invasive property of tumor cells by targeting the upstream signaling cascades that drive EMT initiation and progression (92–94).

Several signaling pathways involved in EMT regulation appear to be affected. The TGF-β/Smad pathway, a central regulator of EMT, may be modulated by the flavonoid isobavachalcone, which inhibits TGF-1-induced phosphorylation of ALK5, ERK, and Smad, and reduces the expression of Snail, while restoring the expression of the epithelial marker E-cadherin in non-small cell lung cancer models (95). Similarly, crude PCL extracts and bakuchiol are associated with the modulation of EMT-related signaling, including NF-kB/Snail pathways and TGF-β receptor-related mechanisms (TGFBR1 and ACVR1B) (96–98). Other pathways, including Notch and Wnt/β-catenin signaling, are two other pathways governing EMT. Psoralidin suppresses Notch-1 signaling and modulates downstream EMT-related factors such as β-catenin and Slug, while increasing E-cadherin expression in breast and prostate cancer models (23, 99, 100). Bakuchiol is similarly associated with reduced Notch3 and Snail expression, inhibiting migration across different cancer lines (97, 98, 101). Furthermore, the coumarin component psoralen influences Wnt/β-catenin signaling through the upregulation of Axin-2, thereby reducing the expression of downstream targets, including Fra-1, impacting EMT-related signaling (102).

At the level of EMT-associated transcription factors and effector proteins, compounds such as 8-methoxypsoralen (8-MOP) and corylin modulate the expression of epithelial markers (E-cadherin) as well as mesenchymal markers (N-cadherin and vimentin) and MMP-9 in murine lung metastasis models (103). Corylin also downregulates the proto-oncogene c-Myc, subsequently modulating EMT markers to inhibit oral squamous cell carcinoma migration (104). Recent evidence also highlights the ability of corylin to modulate non-coding RNA axes, such as miR-34c/LINC00963 and RAD51-AS1, thus inhibiting breast and liver cancer dissemination in cellular assays (105–107).

#### 4.4.2 Modulation of the TME

The TME supports metastatic progression. PCL-derived compounds influence components of this microenvironment, including angiogenesis and extracellular matrix (ECM) remodeling. Psoralidin significantly reduces intratumoral expression of the angiogenesis-related marker CD31 and vascular endothelial growth factor (VEGF) in breast cancer models (77). In addition, Angelicin suppresses the activation of cancer-associated fibroblasts (CAFs), downregulating markers like FAPα and α-SMA, thereby indirectly inhibiting EMT by disrupting the metastatic stromal environment in Lewis lung cancer xenografts (108). Extracellular matrix degradation, a key step in tumor invasion, is mediated in part by matrix metalloproteinases (MMPs). Isobavachalcone and angelicin downregulate the expression and activity of gelatinases MMP-2 and MMP-9 in oral carcinoma cells, as well as in animal models of lung and breast cancer metastasis (39, 45, 109), suggesting a potential role in limiting invasive behavior.

Overall, PCL-derived compounds influence multiple processes associated with tumor invasion and metastasis, including EMT-related pathways, tumor–stroma interactions, and extracellular matrix remodeling, as shown in **Figure 5**. These findings suggest that modulation of both tumor-intrinsic and microenvironmental factors may contribute to the observed biological effects. From a translational medicine perspective, PCL components are uniquely suitable for adjuvant therapies aiming at preventing post-operative recurrence and managing advanced, disseminated diseases.

**Figure 5:**
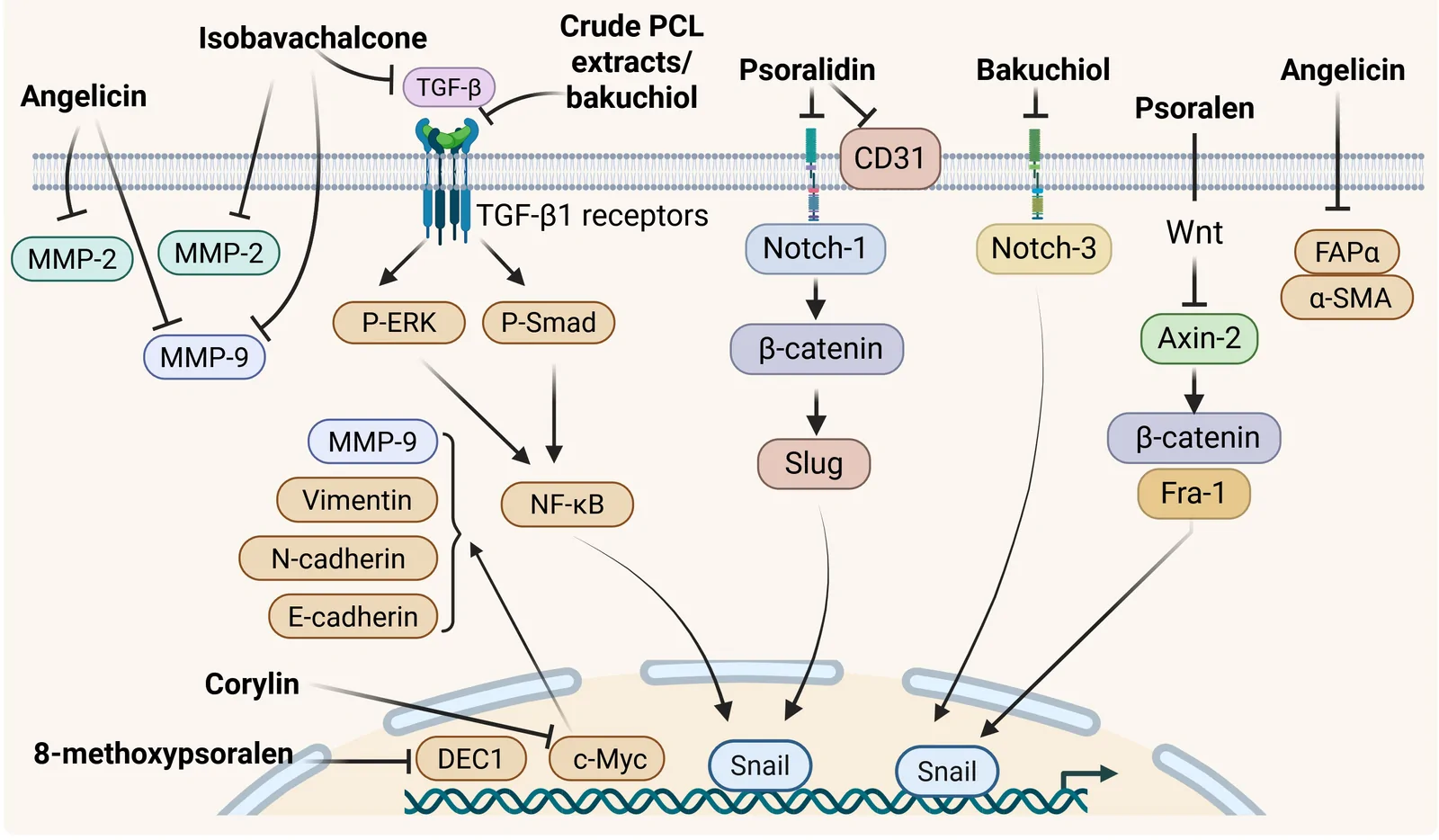
Suppression of tumor invasion and metastasis. Created in BioRender. Leah, R. (2026) https://BioRender.com/dyggx8f.

### 4.5 Overcoming tumor multidrug resistance

#### 4.5.1 Modulation of classical resistance mechanisms

Multidrug resistance remains a major clinical challenge in oncology and is associated with multiple mechanisms, including increased drug efflux, apoptotic evasion, enhanced DNA repair, and tumor microenvironmental adaptation. PCL-derived compounds influence some of these processes, including drug efflux and apoptotic signaling.

Overexpression of P-glycoprotein (P-gp/ABCB1) represents one of the most well-characterized mechanisms of multidrug resistance. Psoralen inhibits P-gp expression (110) at the transcriptional level, suppresses ABCB1 gene expression to reduce P-gp protein synthesis (111), sterically hinders P-gp ATPase activity, and blocks energy-dependent drug efflux, thereby restoring chemosensitivity in resistant lines (93). Isobavachalcone also interacts with membrane-associated transport processes, which affect P-gp-mediated drug transport (112).

In addition, resistance to apoptosis is a key feature of multidrug-resistant tumors characterized by downregulating death receptors or upregulating anti-apoptotic shields. Psoralidin and isobavachalcone enhance the sensitivity of resistant cells to TRAIL-induced apoptosis by upregulating the expression of the death receptor TRAIL-R2 (113, 114). Similarly, neobavaisoflavone is associated with restoration of TRAIL sensitivity in glioma models (115), while bakuchiol amplifies TRAIL signaling through the ROS/JNK axis (116). These observations suggest a potential role in modulating apoptotic responses, rendering tumors vulnerable to standard chemotherapeutics, although further validation is required.

#### 4.5.2 Modulation of emerging resistance mechanisms

In addition to classical multidrug resistance pathways, PCL-derived compounds affect processes associated with tumor recurrence and therapeutic resistance, including cancer stem cell characteristics and epigenetic regulation. Isobavachalcone enhances the sensitivity of ER-positive breast cancer to paclitaxel by the downregulation of CD44 expression, a marker associated with cancer stem cell-like properties (117). These findings suggest that modulation of gene regulatory networks may contribute to altered drug response, although the underlying mechanisms require further investigation. Psoralen enhances chemosensitivity by upregulating miR-196a-5p, which subsequently suppresses the HOXB7-HER2 oncogenic axis (118). This reveals the ability of PCL compounds to reverse the resistant phenotypes by modulating non-coding RNA networks.

Overall, PCL-derived compounds affect multiple mechanisms associated with multidrug resistance, including drug efflux pump function and cellular regulatory pathways, such as restoring intrinsic apoptotic pathway sensitivity, interference with DNA damage repair and metabolism to sensitize cells to specific drugs, further degrading key resistance proteins and modulating tumor stemness. This multi-pronged approach explains the clinical reality of resistance, which is rarely driven by a single mechanism. Consequently, PCL-derived compounds may represent a useful approach for the development of next-generation chemosensitizers and adjuvant therapies capable of destroying complex resistance networks. However, most of the available evidence is derived from preclinical studies, and further investigation is necessary to determine the clinical relevance of these findings.

## 5 Integrated antitumor strategies

Combinations of PCL-derived compounds with conventional or targeted agents have been explored in preclinical studies. For instance, daidzein combined with the selective estrogen receptor modulator centchroman significantly enhances the cytotoxic effect on breast cancer cells through the modulation of the PI3K/Akt/mTOR axis, while simultaneously preserving healthy cells (119). Similarly, co-delivery strategies using nanoparticles or micelles have been investigated to improve pharmacokinetics and therapeutic efficacy, such as the combination of isobavachalcone (an AKT inhibitor) with BIBR1523 (a telomerase inhibitor), achieving synergistic tumor regression and improved pharmacokinetics in liver cancer models (120). The co-delivery of bakuchiol and docetaxel in tumor-microenvironment-responsive micelles not only achieves a specific accumulation in ovarian cancer tissue but also triggers synchronized release, significantly potentiating the pro-apoptotic signaling (121).

PCL-derived compounds influence the sensitivity to conventional chemotherapeutic agents through various mechanisms, highlighting a sensitization based on mechanistic complementarity rather than non-specific additive toxicity. For example, bavachalcone (122) enhances the response to gemcitabine, potentially through the modulation of iron metabolism and growth factor signaling pathways involving the transferrin receptor and epidermal growth factor receptor (EGFR). It amplifies gemcitabine-induced DNA damage stress by disrupting mitochondrial iron metabolism and cellular respiration. This combination regimen demonstrates superior therapeutic efficacy compared to monotherapy in patient-derived bladder cancer xenografts (122). Bavachin increases the sensitivity to paclitaxel in ovarian cancer by interfering with microtubule dynamics or converging survival pathways (28). Daidzein enhances the effects of cisplatin in oral squamous cell carcinoma to significantly inhibit the metastatic properties (123). Gefitinib functions as an EGFR-tyrosine kinase inhibitor that arrests tumor proliferation by competitively blocking the catalytic domain. Daidzein restores sensitivity and synergistically inhibits tumor growth with gefitinib in lung adenocarcinoma models by modulating the ROS/ASK1/JNK and EGFR-STAT/AKT/ERK signaling axes (124). PCL-derived compounds represent potential new strategies in estrogen receptor-positive breast cancer refractory to endocrine therapy. Corylin restores tamoxifen sensitivity by regulating the OAS1/miR-22-3p/SIRT1 axis (125). Bergapten (5-methoxypsoralen) promotes the ubiquitin-proteasome degradation of the estrogen receptor itself, which is the driver of resistance (126). This demonstrates multi-level intervention, ranging from signal modulation to direct target protein clearance.

In modern oncology, PCL provides adjuvant value by mitigating chemotherapy-induced multiorgan toxicity, thereby broadening the therapeutic window. Daidzein attenuates cardiotoxicity induced by doxorubicin by activating the SIRT3/FOXO3a axis to restore mitochondrial energetics (127), while also mitigating paclitaxel-induced neuropathic pain (128). Psoralidin also exerts cardioprotection through the SIRT1/PPARγ pathway (129) and alleviates cisplatin-induced hepatorenal injury (130). Corylin exerts hepatoprotection by inducing UGT1A1 and activating PPARβ/δ and AhR receptors (131). These findings suggest the potential combination of PCL with specific organ-protective effects based on the primary dose-limiting toxicity of a patient’s chemotherapy regimen, enabling personalized protective strategies.

Certain compounds have been investigated in the context of cancer-associated cachexia, which is a metabolic disorder involving multiple organs. Daidzein and corylifol A attenuate skeletal muscle atrophy by inhibiting the Glut4/AMPK/FoxO and TAOK1/p38 MAPK/FoxO3 signaling pathways, and downregulating protein degradation processes (132, 133). Corylifol A also directly targets TAOK3 in pancreatic cancer cells to inhibit tumor growth, thereby achieving a dual improvement of cachexia (134). This ability to concurrently attack the tumor and preserve host tissue highlights the unique advantage of natural multi-component agents in managing complex systemic pathologies.

Recent preclinical studies have developed advanced nanodelivery systems to overcome the inherent trade-offs between the low bioavailability and dose-dependent toxicity associated with monotherapy, including self-microemulsifying formulations, chitosan-coated bilosomes, gold nanoparticles, and polymeric scaffolds (135–138).

These nanocarriers optimize physicochemical properties, enhance intestinal permeability, and promote tumor-targeted accumulation, thereby improving local efficacy without simply increasing systemic doses and potentially alleviating dose-limiting toxicity.

Importantly, preclinical strategies combining PCL compounds with chemotherapy, targeted therapy, or radiotherapy further maximize antitumor potency and reduce drug resistance. Nanotechnology-based co-delivery systems enable the synchronized release of multiple agents and significantly improve preclinical therapeutic outcomes.

However, despite these promising preclinical findings, the clinical translation of both nanoformulations and combination regimens is hindered by unresolved formulation and regulatory challenges. First, the industrial-scale manufacturing of nano-botanical preparations presents challenges in maintaining drug-loading stability, encapsulation efficiency, and batch-to-batch consistency. Second, the co-encapsulation of multiple phytochemicals and synthetic therapeutic agents further increases formulation complexity and complicates quality-control. Third, unified industrial standards and dedicated regulatory guidelines for multicomponent botanical nanomedicines remain absent from current FDA and NMPA regulatory frameworks. Collectively, these bioavailability limitations, dose-dependent risks, formulation bottlenecks, and regulatory gaps hinder the large-scale clinical development of PCL-derived nanotherapeutics and combination strategies.

## 6 Critical evaluation

A critical evaluation of the available evidence indicates that TME reprogramming is supported by some of the most robust and translationally relevant findings. For example, PD-L1 and TME-related modulation has been demonstrated in *in vivo* models including LLC (75) and 4T1 tumor-bearing mice (82), while key mechanisms like ICD have been demonstrated *in vivo* in hepatocellular carcinoma models (67). In contrast, although cellular apoptosis and cell cycle arrest dominate the existing mechanistic literature, most of these classical antitumor mechanisms have been validated only *in vitro* and lack sufficient *in vivo* confirmation (16–18, 29, 33).

Notably, several core antitumor mechanisms of PCL-derived compounds exhibit high reproducibility, having been validated in multiple independent studies and diverse experimental models. Isobavachalcone consistently suppresses MMP-2/MMP-9-mediated metastasis in tongue squamous cell carcinoma and breast cancer models (39, 45). Accumulating evidence also supports DHODH as a direct target of isobavachalcone (44, 68). Nevertheless, most of the proposed molecular mechanisms remain supported by evidence from individual research groups and lack cross-laboratory validation. Classical antitumor events including bakuchiol-induced S-phase arrest (18, 65) and psoralidin-mediated ROS-dependent mitochondrial damage (19–23) are currently limited to *in vitro* observations, with insufficient verification in immunocompetent *in vivo* tumor models.

Apparently divergent pharmacological observations also deserve careful consideration. Psoralidin exhibits different biological effects across independent studies, with contradictory findings reported in the literature (130, 139). These differences may arise from variations in experimental conditions, including compound dose, treatment duration, cell type, animal model, and experimental context.

The translational relevance of the available evidence depends substantially on the experimental model used. Most *in vitro* studies reflect only the direct effects of compounds on isolated tumor cells and cannot reproduce the complexity of the immune microenvironment. Computational target predictions provide preliminary hypotheses but require functional experimental validation. For immunomodulatory and TME-targeted mechanisms of PCL-derived compounds, results obtained using immunocompetent syngeneic tumor models are particularly informative because these models retain an intact host immune system. In contrast, cell-line-derived xenograft and PDX models are useful for assessing intrinsic tumor-cell phenotypes, such as apoptosis and cell cycle arrest, but their utility for studying antitumor immunity is limited because recipient mice are generally immunodeficient. Evidence generated using syngeneic, cell line-derived xenograft and PDX models should therefore be regarded as complementary rather than hierarchically superior, with their relevance interpreted according to the specific research question and experimental system.

## 7 Hepatotoxicity

Clinical studies indicate that PCL-associated hepatotoxicity typically emerges after a median of 29 days of continuous administration, and is often associated with oral doses of 2.4-4.8 g/day (140). Notably, some of the bioactive components contributing to the antitumor effects of PCL may also contribute to idiosyncratic liver injury (141). However, the boundaries between the minimum doses required for antitumor activity and the thresholds associated with hepatotoxicity remain poorly defined for individual purified compounds (7). These compounds can induce oxidative damage and ER stress. For instance, *in vitro* studies have shown that isobavachalcone (IC_50_ = 155.9 μM) induces severe mitochondrial dysfunction in hepatocytes, leading to apoptosis through ROS accumulation (142). Psoralen (80 mg/kg) induces acute ER stress, characterized by the significant upregulation of key markers, inflammatory infiltration, and vacuolar degeneration in liver tissues (143).

Several apparently divergent findings remain unresolved. Although ROS generation and ER stress induce apoptosis in malignant cells (19, 28, 30), these mechanisms may also contribute to hepatotoxicity and necrosis (144). Psoralidin induces liver injury (139). Conversely, a previous study showed that psoralidin mitigates cisplatin-induced hepatic and renal injury (130). Such discrepancies are rarely discussed in existing studies and may be attributable to variations in dose, treatment duration, cell or animal model, experimental context, and the presence or absence of co-administered agents.

In addition to causing direct cellular injury, PCL-induced hepatotoxicity is further exacerbated by metabolic interference. On the one hand, psoralen and 8-MOP significantly interfere with bile acid transporter expression, disrupting bile acid homeostasis and inducing cholestatic liver fibrosis (145, 146). On the other hand, psoralen and isopsoralen significantly disrupt the balance of branched-chain amino acids, aromatic amino acids, and sphingolipids in the liver (147, 148).

Individual susceptibility to PCL-associated hepatotoxicity may be related to genetic variation in drug-metabolizing enzymes, pre-existing liver disorders, and concomitant medication use. Although herbal processing and combination with other agents have been proposed as strategies for mitigating hepatotoxicity, evidence regarding their suitability for long-term anticancer administration remains insufficient. Defining safe therapeutic windows is therefore essential for advancing PCL constituents toward oncological applications.

## 8 Pharmacokinetics and clinical translation of PCL.

From a pharmacokinetic perspective, the major bioactive constituents of PCL, including psoralen, isopsoralen, and bakuchiol, exhibit poor aqueous solubility and variable gastrointestinal absorption, leading to low oral bioavailability (135, 149). In cancer therapy, these properties prevent the compounds from reaching and maintaining effective therapeutic concentrations at the tumor site. Conversely, increasing the administered dose to achieve sufficient antitumor activity may increase the risk of severe systemic toxicity, including the well-documented hepatotoxicity associated with *Psoralea* extracts, thereby narrowing the therapeutic window.

Research on the anticancer activity of PCL is currently dominated by preclinical cell and animal experiments. Systematic human pharmacokinetic studies, registered oncology clinical trials, and large-scale population-based observational studies specifically examining the anticancer effects of PCL remain scarce. Only a small number of exploratory, non-registered clinical observations have been reported. Representative examples include preliminary attempts to use psoralen capsules to reverse multidrug resistance in acute leukemia and compound herbal formulations containing PCL to manage bone metastasis from breast cancer (150, 151). These early reports lack standardized protocols, rigorous control groups, and long-term safety monitoring, and therefore do not constitute robust evidence from registered clinical trials or formal population-based observational studies.

Several interrelated factors may account for the limited availability of high-quality human studies. First, PCL has long been used clinically for dermatological and orthopedic disorders, whereas its anticancer potential represents an emerging research direction, and dedicated human studies on malignancies have not yet been widely initiated. Second, PCL contains complex mixtures of phytochemicals for which unified quality control standards have not been established, creating substantial obstacles to meeting the standardization requirements for oncology clinical trials. Third, the established hepatotoxic risks of PCL-derived compounds may discourage the initiation of long-term human intervention trials for cancer treatment. Fourth, PCL is commonly administered as a component of multi-herb formulations in clinical practice. Isolating its independent antitumor effect is therefore methodologically challenging, restricting the design and implementation of high-quality population-based observational studies.

One promising translational approach is X-ray psoralen activated cancer therapy (X-PACT), which uses synthetic methoxsalen as an intratumoral prodrug and has entered Phase I clinical trials for advanced solid tumors (ClinicalTrials.gov ID: NCT04389281 and NCT06836648). Importantly, methoxsalen is a chemically synthesized psoralen analog rather than a natural compound extracted directly from PCL.

Collectively, several critical barriers hinder the translation of laboratory findings into clinical oncology. Poor solubility and low systemic bioavailability necessitate the development of advanced delivery strategies, such as nanocarriers and local activation approaches, to achieve tumor-selective drug exposure. Safety concerns, including hepatotoxicity and nephrotoxicity, require comprehensive toxicokinetic assessment to define reliable therapeutic windows. In addition, most existing studies focus on individual compounds or signaling pathways, while interactions among different PCL constituents remain insufficiently characterized.

## 9 Conclusion and future perspectives

Accumulating preclinical evidence indicates that *Psoralea corylifolia* L. (PCL) is a valuable source of bioactive phytochemicals with anticancer potential. Its major constituents, including coumarins, flavonoids, and monoterpenoids, influence multiple biological processes associated with tumor progression, such as apoptosis, cell cycle, autophagy, tumor immune microenvironment, metastasis, metabolic reprogramming, and chemoresistance. These effects involve the modulation of key signaling pathways, including ATM/ATR (62), PI3K/AKT (34), MAPK (46), NF-κB (25), and STAT3 (33), as well as the endoplasmic reticulum stress-related signaling (IRE1α-XBP1/JNK) (29), PERK-eIF2α-CHOP (17), and TGF-β/SMAD (95), as shown in **Table 1**. The ability of specific components to reverse multidrug resistance by inhibiting P-glycoprotein function (112) offers a promising strategy to overcome clinical challenges.

**Table 1:** Molecular mechanisms of PCL in oncology.

Mechanism category	Compound name	Core target/signaling pathway	Key biological effect	Experimental model category	Specific experimental model	Reference
Mechanisms of tumor cell death	Psoralen	p53-mediated mitochondrial apoptotic pathway	Induces tumor cell apoptosis	*In vitro*	Human hepatocellular carcinoma SMMC-7721 cell	(16)
		PERK-eIF2α-ATF4-CHOP, ATF6-CHOP ER stress pathway	Induces ER stress-mediated tumor cell apoptosis	*In vitro*	Human hepatocellular carcinoma HepG2 cell line	(17)
	Bakuchiol	Mitochondria-mediated intrinsic apoptotic pathway	Induces intrinsic apoptosis in tumor cells	*In vitro*	Human breast cancer MCF-7 and MDA-MB-231 cell lines	(18)
	Psocorylins R	Mitochondria-mediated intrinsic apoptotic pathway	Induces tumor cell apoptosis	*In vitro*	Human breast cancer MCF-7 cell	(12)
	Psoralidin	JNK1/2-mediated mitochondrial apoptotic pathway	Induces caspase-dependent tumor cell apoptosis	*In vitro*	Human colorectal cancer HT-29 and HCT-116 cell lines	(19)
		Death receptor (DR)-mediated extrinsic apoptotic pathway	Induces tumor cell apoptosis	*In vitro*	Human prostate cancer PC-3 cell line	(20)
		NF-κB-mediated mitochondrial apoptotic pathway	Induces tumor cell apoptosis	*In vitro*	Human colon cancer SW480 cell line	(21)
		Mitochondria-mediated intrinsic apoptotic pathway, death receptor-mediated extrinsic apoptotic pathway	Induces tumor cell apoptosis through dual apoptotic pathways	*In vitro*	Human hepatocellular carcinoma HepG2 cell line	(22)
		Mitochondria-mediated intrinsic apoptotic pathway	Induces mitochondrial dysfunction and caspase-dependent apoptosis	*In vitro*	Human prostate cancer PC-3 and C4-2B cell lines	(23)
	Astragalin	SOX10-mediated mitochondrial apoptotic pathway	Induces tumor cell apoptosis	*In vitro*	Human melanoma A375P and SK-MEL-2 cell lines	(24)
		Mitochondria-mediated intrinsic apoptotic pathway, death receptor-mediated extrinsic apoptotic pathway	Induces caspase-dependent tumor cell apoptosis	*In vitro*	Human colon cancer HCT116 cell line	(25)
	Angelicin	DUSP6/c-MYC signaling pathway	Induces apoptosis	Mixed multi-model evidence	Human oral squamous cell carcinoma HSC-3 and HSC-4 cell lines; human oral squamous cell carcinoma xenograft model	(26)
	Psoralen	Mitochondria-mediated intrinsic apoptotic pathway	Induces p53-independent tumor cell apoptosis	*In vitro*	Human cutaneous T-cell lymphoma MyLa and HuT-78 cell lines	(27)
	Bavachin	Mitochondrial apoptotic pathway, ER stress pathway, MAPK signaling pathway	Induces caspase-dependent tumor cell apoptosis	*In vitro*	Human ovarian cancer ES2 and OV90 cell lines	(28)
	Psoralen	IRE1α-XBP-1s ER stress pathway	Induces ER stress response and subsequent tumor cell apoptosis	*In vitro*	Human hepatocellular carcinoma SMMC7721 cell line	(29)
		ER stress-mediated apoptotic pathway	Induces ER stress-associated tumor cell apoptosis	*In vitro*	Human osteosarcoma MG-63 and U2OS cell lines	(30)
	Isobavachalcone	TrxR1/ROS-mediated apoptotic pathway	Induces ROS-mediated apoptosis and lethal ER stress	*In vitro*	Human prostate cancer PC-3 cell line	(31)
		ERKs/RSK2 signaling axis, mitochondrial apoptotic pathway	Induces liver cancer cell apoptosis	*In vitro*; In silico/computational analysis	Human hepatocellular carcinoma HepG2 and Hep3B cell lines	(32).
	Corylin	STAT3 signaling pathway, mitochondrial apoptotic pathway	Triggers caspase-dependent tumor cell apoptosis	*In vitro*	Human ovarian cancer SKOV3 cell line	(33)
	Bakuchiol	PI3K/AKT signaling pathway, MAPK signaling pathway, mitochondrial apoptotic pathway	Triggers mitochondria-dependent tumor cell death	*In vitro*	Human gastric cancer NUGC3 cell line	(34)
	8-methoxypsoralen	p53 signaling pathway, PI3K/ERK2/STAT3 signaling pathway, mitochondrial apoptotic pathway	Induces caspase-3-mediated tumor cell apoptosis	*In vitro*	Human gastric cancer SNU1 cell line	(35)
		PI3K/AKT signaling pathway, mitochondrial apoptotic pathway, death receptor-mediated extrinsic apoptotic pathway	Induces caspase-dependent tumor cell apoptosis	*In vitro*	Human neuroblastoma SK-N-AS cell line; human colon cancer SW620 cell line	(36)
	Angelicin	TRAIL-mediated apoptotic pathway	Induces tumor cell apoptosis	*In vitro*	Human renal carcinoma Caki cell line	(37)
		PI3K/AKT signaling pathway, mitochondrial apoptotic pathway	Induces dose- and time-dependent tumor cell apoptosis	*In vitro*	Human hepatocellular carcinoma HepG2 and Huh-7 cell lines	(38)
	Isobavachalcone	PI3K/AKT signaling pathway, MAPK/ERK signaling pathway, mitochondrial apoptotic pathway	Triggers typical apoptotic morphological changes in tumor cells	*In vitro*	Human tongue squamous cell carcinoma Tca8113 cell line	(39)
		AKT/GSK-3β/β-catenin signaling pathway; mitochondrial apoptotic pathway	Induces mitochondria-dependent tumor cell apoptosis	*In vitro*	Human colorectal cancer HCT116 and SW480 cell lines	(40)
	5-methoxy psoralen (Bergapten)	PI3K/AKT signaling pathway	Promotes tumor cell apoptosis	*In vitro*; In silico/computational analysis	Human non-small cell lung cancer NCI-H1975, H1299 and H460 cell lines	(41)
		PI3K/AKT/GSK-3β signaling pathway, mitochondrial apoptotic pathway	Induces tumor cell apoptosis through mitochondrial pathway	*In vitro*	Human papillary thyroid carcinoma BCPAP cell line	(42)
	Bakuchiol	Hck/Blk-MEK/ERK/AKT/p70S6K signaling cascades	Exerts chemopreventive activity through the inhibition of neoplastic cell transformation	*In vitro*	Human epithelial carcinoma A431 cell line	(43)
	Isobavachalcone	DHODH-mediated *de novo* pyrimidine biosynthesis pathway, mitochondrial apoptotic pathway	Induces tumor cell apoptosis and myeloid differentiation	*In vitro*	Human acute myeloid leukemia HL60 cell line	(44)
		SIRT2/α-tubulin interaction axis	Induces tumor cell apoptosis	Mixed multi-model evidence	Human breast cancer MCF-7 and MDA-MB-231 cell lines; MDA-MB-231 xenograft model	(45)
	Bavachin	MAPK/Gadd45a signaling pathway	Exerts pro-apoptotic effect in tumor cells	*In vitro*	Human colorectal cancer cell lines	(46)
	Bakuchiol	AMPK/AKT/mTOR pathway, autophagy cascade	Induces autophagy, inhibits hepatocellular carcinoma cell proliferation	*In vitro*	Human hepatocellular carcinoma HepG2 cell line	(47)
	Isobavachalcone	AMPK/AKT/mTOR autophagy pathway	Inhibits NSCLC cell proliferation, induces autophagy	*In vitro*; In silico/computational analysis	Human non-small cell lung cancer cell lines	(48)
	Bergapten	PTEN/p38MAPK/NF-Y axis, PI3K/AKT/mTOR autophagy pathway	Induces autophagy, suppresses breast cancer cell survival	*In vitro*	Human breast cancer MCF7 and ZR-75 cell lines	(49)
	Psoralidin	ROS-mediated autophagy cascade	Inhibits lung cancer cell proliferation through the induction of autophagic cell death	*In vitro*	Human lung cancer A549 cell line	(50)
		NOX4/ROS-mediated autophagy cascade	Induces protective autophagy, inhibits breast cancer cell proliferation	*In vitro*	Human breast cancer MCF-7 cell line	(51)
	Angelicin	mTOR-independent autophagy pathway	Inhibits malignant behaviors of cervical cancer cells through autophagy inhibition	*In vitro*	Human cervical cancer HeLa and SiHa cell lines	(52)
	Bavachin	STAT3/p53/SLC7A11 axis, ferroptosis pathway.	Induces ferroptosis, inhibits osteosarcoma cell viability	*In vitro*	Human osteosarcoma MG63 and HOS cell lines	(55)
	Bavachin	Nrf2/HO-1 pathway, ferroptosis cascade	Induces ferroptosis, suppresses HCC malignant phenotypes	*In vitro*	Human hepatocellular carcinoma Huh-7 and HepG2 cell lines	(56)
	Bavachin	MAPK/STAT3 pathway, ferroptosis cascade	Inhibits laryngopharyngeal cancer growth, induces ferroptosis	*In vitro*	Human laryngopharyngeal cancer Tu212 and FaDu cell lines	(57)
	Psoralidin	ROS/caspase-3/Gasdermin E, ROS/NLRP3/Gasdermin D, pyroptosis pathways	Induces pyroptosis in HCC cells and macrophages	Mixed multi-model evidence	Human hepatocellular carcinoma HepG2 cell line, mouse hepatocellular carcinoma Hepa1–6 cell line; HCC mouse model	(58)
	Corylin	G0/G1 phase cell cycle arrest (cyclin D1/p-Rb)	Induces G0/G1 phase cell cycle arrest	*In vitro*	Human ovarian cancer SKOV3 cell line	(33)
	Psoralen	G1 phase cell cycle arrest (cyclin D1/cyclin E1)	Induces G1 phase cell cycle arrest	*In vitro*	Human hepatocellular carcinoma SMMC7721 cell line	(29)
	Daidzein	G1/S phase cell cycle arrest, TMEM16A calcium-activated chloride channel	Induces G1/S phase cell cycle arrest	*In vitro*	Mouse lung adenocarcinoma LA795 cell line	(59)
		G1/S phase cell cycle arrest (cyclin D1/CDK4, cyclin A/CDK2)	Induces cell cycle arrest	*In vivo*	Diethylnitrosamine (DENA)-induced HCC rat model	(60)
	Angelicin	G1 phase cell cycle arrest, YAP/β-catenin signaling pathway	Induces G1 phase cell cycle arrest	Mixed multi-model evidence	Human glioblastoma cell lines; GBM subcutaneous xenograft nude mouse model and intracranial orthotopic syngeneic GBM mouse model	(61)
	Bavachinin	G2/M phase cell cycle arrest (ATM/ATR-CHK1/CHK2-CDC25C-CDC2)	Induces G2/M phase cell cycle arrest	Mixed multi-model evidence	Human small cell lung cancer H1688 cell line; SCLC xenograft mouse model	(62)
		G2/M phase cell cycle arrest (p38 MAPK-p21Waf1/Cip1)	Induces G2/M phase cell cycle arrest in NSCLC cells	*In vitro*	Multiple human non-small cell lung cancer A549, H23 and HCC827 cell lines	(63)
	Angelicin	G2/M phase cell cycle arrest axis (cyclin B1/cdc2, p21/p27)	Induces G2/M phase cell cycle arrest	*In vitro*	Human triple-negative breast cancer MDA-MB-231 cell line	(64)
	Psoralen	G2/M phase cell cycle arrest (combined with PUVA therapy)	Induces G2/M phase cell cycle inhibition	*In vitro*	Human cutaneous T-cell lymphoma MyLa and HuT-78 cell lines	(27)
	Bakuchiol	S phase cell cycle arrest (ATM/Cdc2/Myt1/Wee1)	Induces S phase cell cycle arrest	*In vitro*	Human breast cancer MCF-7 and MDA-MB-231 cell lines	(18)
		S phase cell cycle arrest	Triggers S phase cell cycle arrest	*In vitro*	Human lung adenocarcinoma A549 cell line	(65)
	Crude PCL extracts	Cyclin D1/CDK4 axis, ERK1/2/GSK3β signaling	Induces cell cycle arrest through the promotion of proteasomal degradation of cyclin D1 and CDK4	*In vitro*	Human colorectal cancer HCT116, SW480, LoVo, HT-29 cell lines	(66)
Reprogramming of the tumor immune microenvironment	Astragalin	NQO2, ROS-mediated ER stress pathway	Induces ICD and enhances PD-L1 blockade	Mixed multi-model evidence	Human liver cancer cell lines, and Hepa1–6 tumor-bearing mouse model	(67)
	Isobavachalcone	STING, T cells/NK cells/dendritic cells immune regulation cascade	Increased infiltration of CD8^+^ T cells, activated CD8^+^CD69^+^ T cells, NK1.1^+^ NK cells, and mature dendritic cells within tumors	Mixed multi-model evidence	C57BL/6J mouse YTN16 syngeneic transplant model; co-culture of human T cells with gastric cancer cells; patient-derived gastric cancer organoids	(68)
	Bakuchiol	AKT/STAT3/PD-L1 signaling pathway	Suppresses PD-L1 expression, modulates anti-tumor immunity, inhibits lung cancer growth	*In vivo*-syngeneic tumor model	Murine Lewis lung carcinoma (LLC) subcutaneous syngeneic tumor-bearing mouse model	(75)
	Isobavachalcone	Tumor immune microenvironment regulation cascade	Modulates anti-tumor immunity, inhibits pancreatic cancer growth	Mixed multi-model evidence	Panc 02 pancreatic cancer cells, RAW 264.7 cells, bone marrow-derived MDSCs; Panc 02 syngeneic pancreatic tumor-bearing mouse model	(76)
	Psoralidin	VEGF/CD31 angiogenic axis, immune regulatory cascade	Inhibits breast cancer growth, modulates anti-tumor immunity	*In vivo*-syngeneic tumor model	4T1 breast cancer syngeneic tumor-bearing Balb/c mouse model	(77)
	Daidzein	NK cell immune regulation cascade	Enhances NK cell anti-tumor immunity	*In vivo*-syngeneic tumor model	4T1 orthotopic breast cancer syngeneic tumor-bearing BALB/c mouse model	(82)
Targeting tumor metabolic reprogramming	Bergapten	Glycolysis, G6PDH, OXPHOS, lipid metabolism cascade	Reprograms energy and lipid metabolism, inhibits breast cancer cell survival	*In vitro*	Human breast cancer MCF7 and ZR75 cell lines	(88)
	Bergapten	LXRα/β-mediated lipid metabolism pathway, PI3K/AKT signaling	Maintains lipid homeostasis, inhibits HCC progression	Mixed multi-model evidence	Human hepatocellular carcinoma HepG2 cell line; NDEA-induced HCC Wistar albino rat model.	(89)
	Astragalin	AMPK/mTOR-mediated aerobic glycolysis axis	Inhibits breast cancer cell proliferation, suppresses aerobic glycolysis	*In vitro*; In silico/computational analysis	Human breast cancer MDA-MB-231 cell line	(90)
		miR125b/HK2-mediated glycolysis/oxidative phosphorylation reprogramming	Suppresses HCC cell proliferation through metabolic reprogramming	Mixed multi-model evidence	Human HCC Huh-7 cell line; Huh-7 xenograft nude mouse model, H22 HCC transplanted Kunming mouse model	(91)
	Bavachin	Mitochondrial energy metabolism pathway	Impairs mitochondrial bioenergetics in tumor cells	*In vitro*	Human ovarian cancer ES2 and OV90 cell lines	(28)
Suppression of tumor invasion and metastasis	Isobavachalcone	ALK5/TGFβ1/Smad/ERK-mediated EMT pathway	Inhibits TGFβ1-induced EMT, suppresses NSCLC cell migration and invasion	*In vitro*	Human lung adenocarcinoma A549 cell line	(95)
	Crude PCL extracts	NF-κB-Snail-mediated EMT pathway	Inhibits LPS-induced EMT, reduces cancer cell migration and invasion	*In vitro*	MDA-MB-231 and SKOV-3 cancer cell lines with LPS-induced EMT phenotype	(96)
	Bakuchiol	Notch3/FASN/TGFBR1/ACVR1B signaling axis	Inhibits breast cancer cell metastasis *in vivo*	Mixed multi-model evidence	Human breast cancer MCF-7 cell line, and zebrafish xenograft model	(97)
	Bakuchiol	TGF-β-mediated EMT pathway (E-cadherin/Snail axis)	Suppresses cancer cell migration, invasion, and *in vivo* lung metastasis	Mixed multi-model evidence	Human lung adenocarcinoma A549, colorectal cancer HT-29, and breast cancer MCF-7 cell lines, and tail vein lung metastasis mouse model	(98)
	Psoralidin	β-catenin/Slug/E-cadherin-mediated EMT pathway	Inhibits migration and invasion of prostate cancer cells through the suppression of EMT	*In vitro*	Human prostate cancer cell line	(23).
		NOTCH1-mediated EMT pathway	Inhibits tumor cell proliferation, EMT, migration, and invasion	*In vitro*	Human breast cancer MDA-MB-231 cell line	(99).
		ALDH/Notch1-mediated EMT pathway	Suppresses breast cancer stem cell-driven tumor growth and metastasis	*In vivo*-xenograft/PDX model	Breast cancer cell xenograft athymic mouse model	(100)
	Bakuchiol	AR/ERβ/NF-κB/MMP-9 signaling pathway	Inhibits prostate cancer cell growth, migration, and invasion	*In vitro*	Human prostate cancer PC-3 cell line	(101)
	Psoralen	Wnt/β-catenin/Fra-1/Axin2 signaling pathway	Suppresses Wnt/β-catenin signaling, inhibits breast cancer malignant phenotypes	*In vitro*	Human breast cancer MCF-7 and MDA-MB-231 cell lines	(102)
	8-methoxypsoralen (8-MOP)	DEC1-mediated EMT signaling pathway	Inhibits HCC cell migration, invasion, and *in vivo* lung metastasis	Mixed multi-model evidence	Human hepatocellular carcinoma HepG2 and SMMC-7721 cell lines, and H22 hepatoma mouse lung metastasis model	(103)
	Corylin	c-Myc-mediated EMT pathway	Inhibits oral squamous cell carcinoma migration and invasion	*In vitro*	Human oral squamous cell carcinoma SAS and OECM1 cell lines	(104)
		p53/miR-34c/LINC00963-mediated EMT pathway	Suppresses breast cancer cell migration and invasion	*In vitro*	Human breast cancer MCF-7 cell line	(105)
		RAD51-AS1/RAD51-mediated DNA damage repair pathway	Inhibits HCC cell migration and invasion	*In vitro*	Human hepatocellular carcinoma HepG2 and Huh7 cell lines	(106)
		GAS5-mediated EMT pathway	Inhibits HCC cell proliferation, migration, invasion, and EMT	*In vitro*	Human hepatocellular carcinoma HepG2 and Huh7 cell lines	(107)
	Psoralidin	Ki-67/CD31/VEGF/IFN-γ/IL-4/FOXp3	Psoralidin inhibits breast tumor angiogenesis	*In vivo*-syngeneic tumor model	4T1 breast tumor-bearing Balb/c mouse model	(77)
	Angelicin	CAF activation pathway	Inhibits CAF activation, suppresses NSCLC growth and metastasis	Mixed multi-model evidence	Murine NIH3T3 and Lewis lung carcinoma cell lines, and LLC xenograft mouse model	(108)
	Isobavachalcone	Akt/ERK/MMP-2/MMP-9 signaling pathway	Inhibits tumor cell migration and invasion	*In vitro*	Human tongue squamous cell carcinoma (TSCC) Tca8113 cell line	(39)
		SIRT2/α-tubulin/Snail/MMP signaling pathway	Suppresses tumor cell migration and invasion	*In vitro*	Human breast cancer MDA-MB-231 cell line	(45)
	Angelicin	ERK/JNK/MMP2/9/E-cadherin signaling pathway	Inhibits NSCLC cell growth and metastasis	Mixed multi-model evidence	Human non-small cell lung cancer A549 cell line, A549 xenograft, and lung metastasis mouse model	(109)
Reversal of tumor multidrug resistance (MDR)	Psoralen	P-gp (MDR1)	Reverses P-gp-mediated multidrug resistance, enhances adriamycin chemosensitivity	*In vitro*	Human breast cancer MCF-7 and MCF-7/ADR cell lines	(110)
		ABCB1	Reverses docetaxel resistance in lung cancer cells	*In vitro*	Human non-small cell lung cancer A549 and A549/D16 cell lines	(111)
		P-gp ATPase	Reverses P-gp-mediated multidrug resistance	*In vitro*	Human breast cancer MCF-7/ADR cell line	(93)
	Isobavachalcone	ABCB1 (P-gp)	Reverses ABCB1-mediated multidrug resistance	*In vitro*	Human colorectal adenocarcinoma HT-29 and HT-29/Dx cell lines, and ABCB1-transfected MDCK cell line	(112)
	Psoralidin	TRAIL-mediated apoptotic pathway	Sensitizes TRAIL-resistant prostate cancer cells to TRAIL-induced apoptosis	*In vitro*	Human prostate cancer LNCaP cell line	(113)
	Isobavachalcone	TRAILR2 (DR5)/TRAIL-mediated apoptotic pathway	Sensitizes HeLa cells to TRAIL-induced apoptosis	*In vitro*	Human cervical cancer HeLa cell line	(114)
	Neobavaisoflavone	DR5/TRAIL signaling pathway	Sensitizes TRAIL-resistant glioma cells to anoikis resistance	*In vitro*	Human glioma U373MG cell line	(115)
	Bakuchiol	ROS/JNK/DR4/DR5/cFLIP signaling pathway	Sensitizes colon cancer cells to TRAIL-induced apoptosis	*In vitro*	Human colon cancer HCT116 and HT-29 cell lines	(116)
	Isobavachalcone	ERα/CD44 signaling pathway	Overcomes paclitaxel resistance in ER-positive breast cancer	Mixed multi-model evidence	Estrogen receptor-positive and paclitaxel-resistant breast cancer cell lines, and xenograft models	(117)
	Psoralen	miR196a5p/HOXB7/HER2 signaling pathway	Reverses cisplatin resistance in gastric cancer	*In vitro*	Human gastric cancer MGC803 and cisplatin-resistant cell lines	(118)

Nevertheless, the translational gap remains substantial. Current research is dominated by preclinical studies, whereas high-quality human pharmacokinetic studies, registered oncology clinical trials, and large-scale observational studies examining the anticancer activity of PCL remain limited. As outlined in Section 8, key obstacles include unfavorable physicochemical properties, low bioavailability, dose-dependent systemic toxicity, undefined therapeutic windows, and an insufficient understanding of multicomponent interactions. Although emerging strategies, such as targeted nanodelivery, combination regimens and local photoactivated therapy, have produced encouraging preclinical outcomes, numerous formulation and translational challenges remain unresolved.

Priorities for accelerating bench-to-bedside translation include the use of multi-omics and network pharmacology approaches to systematically characterize compound–target–effect relationships and synergistic interactions among PCL constituents and between PCL and other herbal medicines. Understudied chemical fractions such as benzofurans and lipids warrant further pharmacological evaluation. Standardized quality control specifications for PCL raw materials and preparations should also be established. Furthermore, conventional *in vitro* and animal models should be complemented by clinically relevant platforms, such as patient-derived tumor models, to improve the predictive value of preclinical data. Ultimately, rigorously designed human studies are required to validate the anticancer efficacy and safety of PCL-derived compounds, and determine their therapeutic potential in clinical oncology.

In addition to oncology-focused research, interdisciplinary studies are warranted to further investigate the translational potential of *Psoralea corylifolia* in dermatological and inflammatory diseases and assess the potential contribution of its pharmacological properties to integrative healthcare.
